# Real-Time Coordination of the Regional Health System During the Pandemic

**DOI:** 10.1017/dmp.2020.501

**Published:** 2020-12-22

**Authors:** Matteo Nocci, Bassam Dannaoui, Francesco Della Corte, Luca Ragazzoni, Francesco Barone-Adesi, Stefano Romagnoli, Angelo Raffaele De Gaudio, Francesca Rubulotta, Maria Teresa Mechi

**Affiliations:** 1 Quality of care and clinical networks Regional Health Department - Tuscany Region, Florence, Italy; 2 CRIMEDIM - Research Center in Emergency and Disaster Medicine, University of Piemonte Orientale, Novara, Italy; 3 Health Science Department, Section of Anesthesia and Critical Care, University of Florence, Azienda Ospedaliero-Universitaria Careggi, Florence, Italy; 4 Technological Innovation in Clinical-Assistance Activities Unit, Azienda Ospedaliero-Universitaria Careggi, Florence, Italy; 5 Anesthesia and Intensive Care Medicine, Imperial College of London, London, UK

**Keywords:** disaster medicine, hospitals, surge capacity

The 2019 coronavirus disease (COVID-19) pandemic is an enormous challenge for health care systems, with great impact on hospital response.^1^ Advanced organization based on hospital alliances and consistent coordination among different levels is essential to handle high influx scenarios and expand surge capacity of the entire health system.^2-4^ This paper describes the experience of the real-time coordination of the Tuscany region, Italy, a network of 43 hospitals with a total availability of approximately 11 000 beds, during the COVID-19 pandemic, through the use of a novel computerized operational tool (COV19-OT). COV19-OT is a web-based platform (developed with php, MySQL, and jQuery protocols) that aims to continuously track the critical components of hospital preparedness and response, especially regarding surge capacity and workloads of single hospitals.

## Methods

COV19-OT is the collection point for all data from regional hospitals and other connected health care facilities (such as bed availability, occupancy surge capacity, equipment, etc.). The tool generates additional information (indicators) related to surge capacity at different levels (single hospital, health care company, regional). In terms of structure, the COV19-OT platform is made up of several different sections, the access to and functions of which depend on the user’s profile and permission. The structure is described in Table 1.

**Table 1.** COV19-OT structure

N	Section	Description	User Profiles and Permissions						
			1	2	3	4	5	6	7
**1**	Health care facilities	List of facilities of regional health care system (eg, hospitals, post-care facilities, rehabilitation, retirement homes, field hospital, etc.)	I - M	I - M	NV	V	NV	NV	NV
**2**	Areas	COVID-19 and non-COVID-19 areas in the health care facilities listed above. Areas are categorized according to treatment level (intensive, subintensive, not-intensive). This section could include ED areas referring to patients waiting for ward admission.	I - M	I - M	NV	I - M	NV	NV	NV
**3**	Resources	Resources available in a specific area. Each single bed is intended as bed with all necessary equipment and staff. Beds are categorized as not-isolated, isolated, and under negative pressure in 4 different categories: 1. Promptly available 2. First-line activable (within 48 hours) 3. Second-line activable (48-120 hours) 4. Third-line additional beds (over 120 hours) The section includes fields for additional resources needed to increase number of beds available, such as critical care resources (ventilators, monitors, etc.).	I - M	I - M	NV	I - M	NV	NV	NV
**4**	Availability	Updated occupancy and availability rate related to beds inserted in section 3. Additional information is required regarding ventilation mode in ICU beds (invasive, non-invasive).	I - M	I - M	NV	I - M	NV	NV	NV
**5**	Dashboard	Information management tool that allows real-time tracking of updated information regarding facilities inserted in section 1; dashboard is structured with a colored layout (Figure 1).	M	V	V	V (p)	V (p)	NV	NV
**6**	Report	Automatically generated twice daily. Information is categorized by organizational level (single hospital, health care company, large areas, regional), COVID-19/non-COVID-19 beds and the following: 1. Capacity: number of beds available for each treatment level 2. Expansion: number of additional beds activable in first, second, and third line 3. Additional resources (needed for extra beds) 4. Occupancy: number of beds occupied for each treatment level, including information on invasive/non-invasive ventilation 5. Surge indicators: • *Relative surge capacity* (percentage increase of COVID-19 beds relative to baseline COVID-19 beds) • *Absolute surge capacity* (percentage increase of COVID-19 beds relative to baseline total beds) • *Hospital workload level/pressure* (percentage of occupied intensive beds relative to total number, including additional beds) • *Intensive/non-intensive* bed occupancy ratio	M - D	V	V - D	V (p)	V	V	V - D
**7**	Graphs	Graphical visualization of all data trends listed in section 6	M	V	V	V (p)	V	V	V
**8**	Technical and logistic	Tracking tool for medical devices and equipment (eg, critical care resources) managed by technical and logistics department available to bolster hospital response	I - V - M - D	I – V - M	NV	NV	NV	I - V - M - D	NV
**9**	File manager	Collection of files on procedures, protocols, regulations, guidelines	I - V - M - D	I - V - M - D	NV	NV	NV	NV	NV
**10**	Contacts	Address book	I - V - M - D	I - V - M - D	NV	V	NV	V	NV

***Notes:*** **User profile**: (1) COV19-OT developer, (2) Regional Operation Centre operators (ROC), (3) Regional Disaster Response coordinators (RDRC), (4) Hospital and health care facility referents* (5) Health care company managers, (6) Technical and logistics department referents, (7) Data managers/Regional civil protection referents

**User permission**: I = insert data; V = view data; V(p) = partial view data; M = modify; D = download data; NV = not visible (*only for own structure/service).

**Figure 1.**
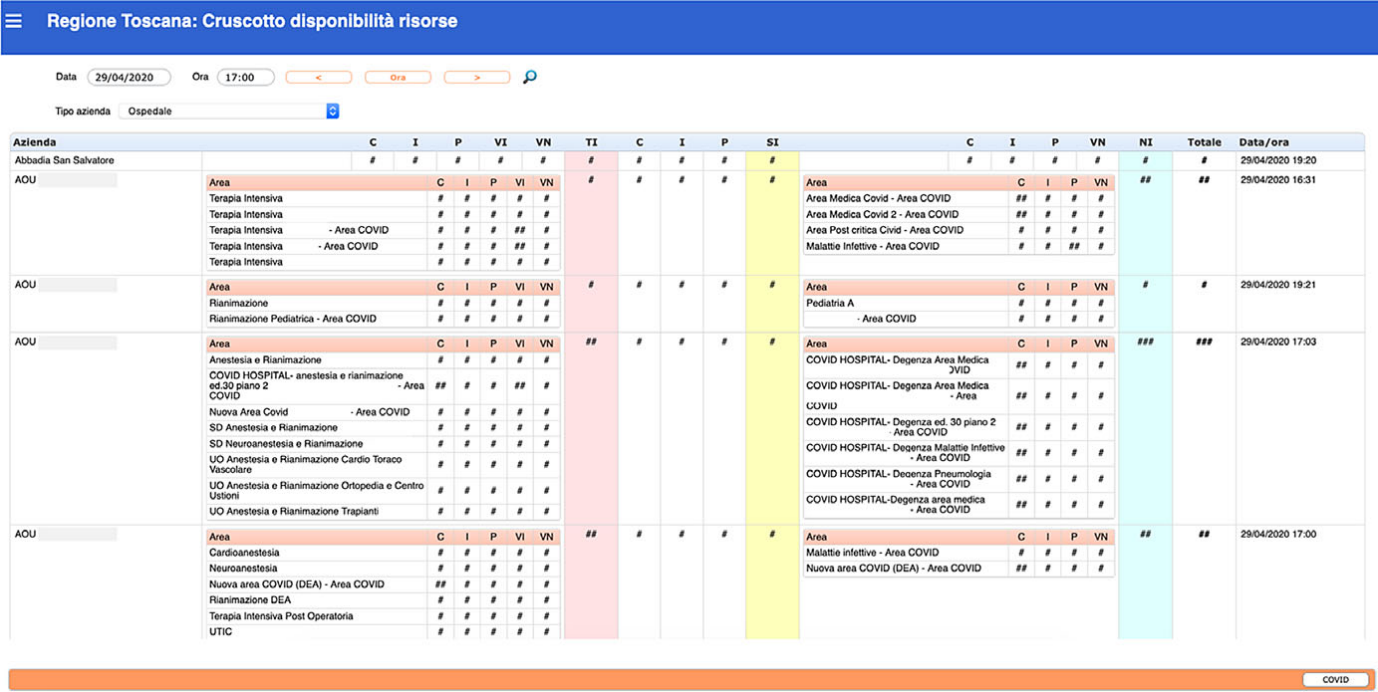
Dashboard *(Italian. Partial view. Numbers are hidden).* Structured with a colored layout, this section tracks, analyzes, and displays information for a single hospital or health care facility. Areas are described for each hospital or health care facility (column 1). For each area, the number of beds is updated at least twice daily, according to level of treatment: • TI = intensive care (total in pink column) • SI = sub-intensive care (total in yellow column) • NI = non-intensive care (total in blue column) Type of location: • C = multi-bed room • I = isolated • P = negative pressure • VI = invasive ventilation; VN = non-invasive ventilation. The “COVID” button changes to a view of the same information regarding COVID-19 occupied beds, specifically.

## Results and Discussion

The assessment of hospital surge capacity and occupancy levels during a pandemic is an organizational requirement,^4,5^ and inter-hospital coordination must be guaranteed in order to enact the necessary operative strategies. In our experience, COV19-OT allows updated, real measurement of additional beds available, as well as the calculation of surge indicators at any level, providing a data set that is not commonly available and is extremely useful during hospital overflows.

COV19-OT provides a precise mapping of COVID-19 and non-COVID-19 areas throughout the regional territory and the relative number of beds. At the operative level, the analysis of surge indicators allows users to activate strategies to balance workloads and avoid single-hospital saturation (eg, prehospital flow diversion or bio-containment medical transfer) and quickly move critical resources (eg, staff, ventilators, monitors). At the planning level, COV19-OT has allowed us to establish escalation or de-escalation strategies at each level (opening or closing of new COVID-19 or non-COVID-19 areas/hospitals) and consequently plan resource allocation based on indicators or critical threshold alerts. More importantly, the COV19-OT has guaranteed a rational approach to decision-making (identifying a critical threshold of hospital pressure on 32 days during which most of the operative strategies were adopted) and a rational approach, by sharing a unique management system based on common operative principles (ie, indicators, threshold, parameters).

The use of the tool could be limited by the need to manually upload data, which increases the risk of errors and referents’ workloads; careful, continuous data checking is necessary. This limitation could be partially remedied with automated filing through an existing admission, discharge, and transfer (ADT) system.

## Conclusion

COV19-OT is a promising tool to consistently assess surge capacity and related indicators in multi-hospital systems, improving real-time hospital coordination during hospital overload scenarios. Additional studies are needed to assess its impact on patient quality of care, outcomes, and health care system benefits.
